# Chronic Granulomatous Disorder–Associated Colitis Can Be Accurately Evaluated with MRI Scans and Fecal Calprotectin Level

**DOI:** 10.1007/s10875-019-00651-2

**Published:** 2019-06-06

**Authors:** David M. Lowe, Philip J. Smith, Fernando Moreira, Sarita Workman, Helen Braggins, Nikolaos Koukias, Matthew S. Buckland, Peter Wylie, Stuart A. Taylor, Charles D. Murray

**Affiliations:** 1 Institute of Immunity and Transplantation, University College London, Royal Free Campus, Pond Street, London, NW3 2QG UK; 2 Department of Clinical Immunology, Royal Free London NHS Foundation Trust, Pond Street, London, NW3 2QG UK; 3 Department of Gastroenterology, Royal Free London NHS Foundation Trust, Pond Street, London, NW3 2QG UK; 4 Department of Gastroenterology, Royal Liverpool and Broadgreen University Hospitals NHS Trust, Prescot St, Liverpool, L7 8XP UK; 5 Department of Immunology, Great Ormond Street Hospital, London, WC1N 3JH UK; 6 Department of Radiology, Royal Free London NHS Foundation Trust, Pond Street, London, NW3 2QG UK; 7 Centre for Medical Imaging, University College London Hospital, University College London, Euston Road, London, NW1 2BU UK

**Keywords:** Non-invasive assessment, inflammatory bowel disease, endoscopy, radiology

## Abstract

**Purpose:**

Colitis is a common and serious complication of chronic granulomatous disorder (CGD) and requires assessment. Colonoscopy is invasive and carries risks of serious complication. We therefore assessed non-invasive monitoring via magnetic resonance imaging (MRI). We also evaluated fecal calprotectin (FCP), the Harvey-Bradshaw index (HBI) clinical score, and serum cytokines.

**Methods:**

We recruited 10 patients with CGD (8 males, mean age 29.6 years), scored a modified HBI, and obtained stool for FCP. The following day we took blood for cytokine measurement via Luminex, performed MR enterography (scored by two independent radiologists using three systems: London score, CDMI, and MaRIA) followed by colonoscopy with disease activity measurement via ulcerative colitis endoscopic index of severity (UCEIS). We assessed patient experience after each investigation and overall preference with follow-up questionnaires.

**Results:**

MRI scores correlated well with colonoscopic gold standard (for London score *R*^2^ 0.91, *p* < 0.0001; for CDMI *R*^2^ 0.83, *p* = 0.0006; for MaRIA *R*^2^ 0.89, *p* = 0.0002). MRI was better tolerated and generally preferred, quicker, and visualized the entire large bowel whereas colonoscopy did not reach the terminal ileum in 3 participants. Elevated FCP accurately differentiated patients with colitis from those without, and log(calprotectin) correlated well with disease activity (*R*^2^ 0.71, *p* = 0.009). Serum interleukin (IL)-12 concentration correlated with colitis activity but IL-1β and TNF did not. Harvey-Bradshaw index did not correlate with colitis activity.

**Conclusions:**

MRI and fecal calprotectin are useful methods for monitoring CGD colitis and should reduce the need for colonoscopy in these patients. IL-12 may represent an appropriate target for treatment.

**Electronic supplementary material:**

The online version of this article (10.1007/s10875-019-00651-2) contains supplementary material, which is available to authorized users.

## Introduction

Chronic granulomatous disorder (CGD) is a primary immunodeficiency caused by mutations in the nicotinamide adenine dinucleotide phosphate (NADPH) oxidase enzyme complex [1]. Affected individuals suffer recurrent infection with bacterial and fungal pathogens but also develop inflammatory complications, most notably a colitis [2]. CGD-associated colitis is distinct from other inflammatory bowel diseases: the distribution of disease often resembles ulcerative colitis [3] but histologically more resembles Crohn’s disease [4], characterized by granulomas and cryptitis. Colitis contributes a significant burden of morbidity in CGD, and treatment can be complex [5].

As with other forms of inflammatory bowel disease (IBD), it is important to monitor the activity of CGD-associated colitis. This is traditionally undertaken by colonoscopy, which is an invasive procedure carrying a risk of perforation and hemorrhage [6]. Furthermore, histological appearances tend to be consistent between patients and do not necessarily inform management; thus, the utility of obtaining biopsies is limited.

More recently, MRI has been shown to be accurate in the diagnosis and monitoring of inflammatory bowel diseases [7–9]. This imaging modality is considered safe and does not involve ionizing radiation. We sought to ascertain whether MRI could accurately assess the colonic appearance in CGD, compared with colonoscopy as a gold standard. We also investigated other non-invasive monitoring techniques. The fecal calprotectin (FCP) level, now well established in other IBD, has recently been suggested to be useful in pediatric CGD-associated colitis [10]. We also assessed a clinical score based on the Harvey-Bradshaw index (HBI) used for Crohn’s disease [11] and blood levels of inflammatory cytokines other than C-reactive protein, focusing on molecules which have pharmacological inhibitors available.

## Materials and Methods

### Recruitment and Ethics

Adult patients (≥ 18 years) with a confirmed diagnosis of CGD were recruited from the national CGD clinic at the Royal Free Hospital, London, or via the UK national CGD specialist nurse. Inclusion and exclusion criteria are listed in Supplementary Table 1. We estimated approximately 30 patients were eligible nationally and recruited 10. The study was approved by the NHS Research Ethics Committee (REC 15/LO/1334) and all participants provided written informed consent.

### Study Design

Patients were clinically assessed, with calculation of a modified Harvey-Bradshaw index (see below), within 1 week of the investigations. A day before the procedures, patients took standard bowel cleansing with Moviprep® (polyethylene glycol). They were provided with containers to collect stool samples for fecal calprotectin (before commencing Moviprep) and investigation of infection (see below) which they brought to hospital on the day of the procedures.

On the day of the investigations, blood was collected for measurement of C-reactive protein and serum cytokines; patients drank up to 2 l of 2% mannitol solution as MRI contrast, the first half between 2.5 and 1.5 h before the scan time (in order to reach the colon) and the second half over the last hour before the scan time (to distend the small bowel). The actual volume ingested was occasionally adjusted according to patient tolerance. Patients then underwent MRI scanning and 10 min afterwards were given a questionnaire regarding tolerability (see Supplementary Appendix 1). They subsequently underwent colonoscopy and after recovery from sedation were given a similar questionnaire pertaining to their experience of colonoscopy (see Supplementary Appendix 2). When fully recovered, patients returned home. One week later, they were sent a follow-up questionnaire regarding tolerability and preferences (see Supplementary Appendix 3).

### MRI Scanning and Scoring Systems

MRI was performed on a Philips (Philips Medical Systems, Eindhoven, Netherlands) 1.5T Ingenia or 1.5T Nova Dual scanner. Table 1 summarizes the sequence of MRI images and adjunctive medications administered. Scoring of MRI images was performed independently by two specialist radiologists with 12 and 17 years of experience in enteric MRI, respectively, blinded to colonoscopy findings. Three validated MRI activity scores were calculated (see Supplementary Appendix 4 for further details):

- The “London” score [12], calculated as 1.79 + 1.34 × mural thickness + 0.94 × mural T2 signal
- The Crohn’s disease MRI index (CDMI) score [12], calculated as mural thickness + mural T2 signal + enhancement + perimural T2 signal
- The magnetic resonance index of activity (MaRIA) score [13], calculated as 1.5 × wall thickness (mm) + 0.02 × relative contrast enhancement + 5 × edema + 10 × ulceration

**Table 1:** Summary of MRI sequences and adjunctive medications used in the study

20 mg intravenous hyoscine butylbromide (unless contraindicated) MRI sequences (Philips 1.5T with body coil): 5 mm coronal and axial SSH-TSE (HASTE) 5 mm coronal and axial BFFE (Tru-FISP) 2.5 mm coronal dynamic pre- and post-contrast (intravenous gadolinium chelate 0.2 mg/kg) THRIVE (VIBE) 2.5 mm axial delayed post-contrast THRIVE (VIBE) 6 mm axial diffusion (0, 50, 600) with ADC map	

*SSH* single shot, *TSE* turbo spin echo, *HASTE* half-Fourier-acquired single-shot turbo spin echo, *BFFE* balanced fast field echo, *FISP* fast imaging with steady state precession, *THRIVE* T1 high-resolution isotropic volume excitation, *VIBE* volumetric interpolated breath-hold examination, *ADC* apparent diffusion coefficient

Scoring was performed on each bowel segment (rectum, sigmoid, splenic flexure, transverse colon, hepatic flexure, caecum, and terminal ileum) and is presented as the average from the two radiologists. A total score and a “colon-only” score (without the terminal ileum) was then calculated for each scoring system.

### Colonoscopy and Scoring System

A standard colonoscopy was performed as far as the terminal ileum (if possible) with sedation and analgesia as required. The severity of colitis was scored as consensus between two endoscopists for each bowel segment (rectum, sigmoid, splenic flexure, transverse colon, hepatic flexure, caecum, and terminal ileum) using the ulcerative colitis endoscopic index of severity (UCEIS) system [14]. This validated system assesses the vascular pattern, evidence of bleeding, erosions, and ulcers. A total score and a “colon-only” score (without terminal ileum) were then calculated. Biopsies were taken from each bowel segment for histopathological assessment, except the transverse colon. From the histological patterns observed, we classified biopsies as “normal” (the presence of pigmented macrophages was permitted), demonstrating acute changes of colitis (cryptitis, crypt abscesses, inflammatory cell infiltrate, and ulceration) or demonstrating chronic quiescent changes only (crypt architectural distortion or granulomas without inflammation).

### Modified Harvey-Bradshaw Index and Patient Questionnaires

The Harvey-Bradshaw index is a clinical index which is used in Crohn’s disease patients to define disease activity [11]. Lacking a specific clinical index for CGD, we utilized the Harvey-Bradshaw score because of similar histology and often similar symptoms to Crohn’s disease. However, we slightly modified the list of complications as CGD patients would not be expected to suffer some conditions specific to Crohn’s. The modified Harvey-Bradshaw scoring is summarized in Table 2.

**Table 2:** Modified Harvey-Bradshaw index

Parameter	Scoring
General well-being	0 = very well 1 = slightly below average 2 = poor 3 = very poor 4 = terrible
Abdominal pain	0 = none 1 = mild 2 = moderate 3 = severe
Liquid stools	Number of liquid stools per day
Abdominal mass	0 = none 1 = dubious 2 = definite 3 = tender
Complications	1 point for each: anal fissure, active fistulae, abscess

### Measurement of Serum C-Reactive Protein, Cytokines, and Markers of Immune Activation

C-reactive protein was measured in patient serum via nephelometry in the hospital diagnostic laboratory. Cytokines (interleukin (IL)-1β, IL-6, IL-12, and tumor necrosis factor (TNF)), markers of immune activation (soluble cluster of differentiation 14 (sCD14)), and endothelial activation (intercellular adhesion molecule-1 (ICAM-1)) were measured via Luminex technology at the Multiplex Core Laboratory, UMC Utrecht, Netherlands. We assigned a value of zero to any undetectable results.

### Measurement of Fecal Calprotectin and Tests for Stool Pathogens

Fecal calprotectin was measured via enzyme immunoassay in the diagnostic laboratory. To exclude infection, diagnostic laboratories performed polymerase chain reaction (PCR) for viruses (adenovirus, norovirus, enterovirus, rotavirus, and sapovirus) and bacteria (*Campylobacter*, *Salmonella*, *Shigella*, *Escherichia coli* O157). *Clostridium difficile* was screened via the glutamate dehydrogenase (GDH) detection test with subsequent testing for toxin and toxin gene if required. Microscopy was performed to exclude helminths or protozoa (including ova and cysts).

## Results

### Patient Characteristics

Table 3 details the patient characteristics. Of the 10 patients recruited, seven had X-linked CGD (gp91^phox^) and there was one patient each with p40^phox^, p47^phox^, and p67^phox^ autosomal recessive CGD. Eight patients were male and the mean age was 29.6 years. All patients were receiving antibiotics and antifungal prophylaxis; four patients were on significant immunosuppression and one further patient was taking mesalazine. Seven patients had a known history of colitis prior to the investigations, and the majority of patients had previously undergone both MRI and colonoscopy.

**Table 3:** Clinical characteristics of participants in the study

ID	Age (years)	Sex	CGD type	Known history of colitis	Immunosuppression	Co-trimoxazole	Itraconazole	Other antimicrobials	Other medication
1	22	M	XL	Y	IV hydrocortisone, Pentasa, hydroxychloroquine	N	Y	Ciprofloxacin, metronidazole	Omeprazole, paracetamol, tramadol
2	27	M	XL	Y	Nil	Y	Y	Nil	Nil
3	39	M	XL	Y	Mesalazine	Y	Y	Nil	Nil
4	24	M	XL	Y	Prednisolone (15 mg daily), Asacol	Y	Y	Nil	Fultium-D3, ferrous fumarate
5	24	F	p67	Y	Prednisolone (25 mg daily), Mezavant (extended release), azathioprine	Y	Y	Nil	Calcichew-D3 forte
6	25	M	XL	Y	Nil	Y	Y	Nil	Nil
7	37	M	XL	N	Nil	N	Y	Ciprofloxacin, doxycycline	Citalopram, candesartan, calcichew-D3 forte, lansoprazole, mebeverine, alendronate
8	35	F	p47	Y	Azathioprine	Y	Y	Nil	Venlafaxine, fexofenadine
9	26	M	p40	N	Nil	Y	Y	Nil	Nil
10	37	M	XL	N	Nil	Y	Y	Nil	Nil

*XL* X-linked, *IV* intravenous, *mg* milligrams

All tests for gastrointestinal infection were negative. Further scores and measurements generated in the study are detailed in Supplementary Table S2. Clinical features of inflammatory bowel disease, defined by the Harvey-Bradshaw parameters, are presented in Supplementary Table S3, and detailed endoscopic findings are included in Supplementary Table S4.

### Completion Rates and Adverse Events in MRI and Colonoscopy

MRI scans were completed successfully in all participants, with no significant adverse events attributed to MRI. The mean (± SD) time recorded for MRI was 27.6 ± 6.6 min. Colonoscopy reached the terminal ileum in seven, caecum in two, and splenic flexure in one patient. All patients received intravenous sedation except one who only received inhaled nitrous oxide (Entonox®). The mean (± SD) time recorded for colonoscopy, regardless of whether the terminal ileum was reached, was 35.7 ± 14.6 min. One participant was briefly hospitalized due to fever, abdominal pain, and rigors on the night after the procedures. No organisms were identified in blood cultures, and the episode was considered to possibly relate to translocation of bacterial products during colonoscopy.

### MRI and Colonoscopy Scores of Colitis Activity Correlate Well

As colonoscopy did not reach the terminal ileum in three patients, we used total UCEIS score and total MRI scores as far as the caecum (*n* = 9) for study outcomes. Overall correlation between the two radiologists was good (*R*^2^ 0.90 for the London score, 0.88 for CDMI, and 0.82 for MaRIA). Results from all scoring systems indicated maximal colitis in the distal colon (Supplementary Fig. 1).

**Fig. 1:**
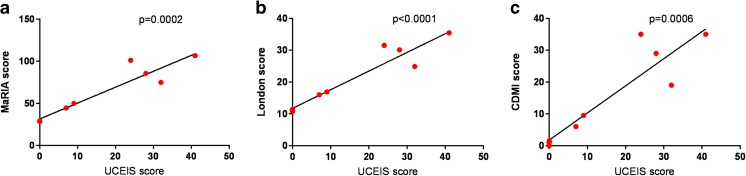
Correlations between endoscopic and MRI scores. **a**–**c** Correlations between the endoscopic UCEIS score and the MRI scoring systems, MaRIA score (**a**), London score (**b**), and CDMI score (**c**). Analysis is based on the total scores for the entire colon (rectum to caecum) and *p* values are derived from Pearson correlation

As detailed in Fig. 1, there was a strong correlation between UCEIS and each of the MRI scores (for London score *R*^2^ 0.91, *p* < 0.0001; for CDMI *R*^2^ 0.83, *p* = 0.0006; for MaRIA *R*^2^ 0.89, *p* = 0.0002). Significant correlations persisted when analysis was restricted only to patients with macroscopic colitis (*n* = 6; for London score *R*^2^ 0.80, *p* = 0.02; for CDMI *R*^2^ 0.66, *p* = 0.05; for MaRIA *R*^2^ 0.75, *p* = 0.03).

We also analyzed correlation between UCEIS and the MRI scores for each bowel segment, as detailed in Table 4. There was a significant correlation for each segment. However, MRI suggested presence of inflammation in the rectum in three out of four patients where colonoscopy was normal. In two of these cases, the MRI was reported as abnormal by only one radiologist and suggested mild disease. However, in one patient, the MRI report from both radiologists was compatible with significant colitis: this patient had previously received treatment for anal cancer and perianal fistulae. In total, five patients had clinical evidence of active perianal disease, and MRI appeared to overinterpret rectal findings compared with colonoscopy in two of these.

**Table 4:** Correlation of UCEIS scores with each of the MRI scores according to bowel segment

Bowel segment	*n*	MaRIA score		London score		CDMI score	
		*R* ^2^	*p* value	*R* ^2^	*p* value	*R* ^2^	*p* value
Rectum	10	0.51	0.02	0.62	0.007	0.68	0.003
Sigmoid	10	0.82	0.0003	0.82	0.0003	0.84	0.0002
Descending colon/splenic flexure	10	0.88	< 0.0001	0.74	0.001	0.77	0.0009
Transverse colon	9	0.52	0.03	0.65	0.009	0.62	0.01
Hepatic flexure/ascending colon	9	0.89	0.0002	0.84	0.0005	0.83	0.0006
Caecum	9	0.64	0.01	0.56	0.02	0.56	0.02
Terminal ileum	7	0.996	< 0.0001	1	N/A	1	N/A

*N/A* not applicable

In one patient’s MRI, one radiologist’s interpretation suggested presence of disease in the transverse colon even though colonoscopy was normal: this patient did have severe macroscopic colitis as far as the splenic flexure and this may therefore represent difference between modalities or investigators in identifying the transition point. There were also six bowel segments across the study (out of 64 examined by colonoscopy) where MRI failed to identify macroscopic colitis: half of these were in the caecum while none were in the sigmoid or rectum. None of these discordant segments had severe colitis (maximal UCEIS score 4). In all segments where there was discordance and available biopsies, histology supported the colonoscopy findings.

### Tolerability and Patient Preferences for MRI Versus Colonoscopy

When asked about the least acceptable and overall worst parts of the procedures (Table 5), participants identified different aspects of MRI including bowel preparation, breath holds, noise, and various forms of pain or discomfort. For colonoscopy, there was near-unanimous reporting that bowel preparation was the least acceptable, and this was also stated by 3 patients as the “overall worst” aspect; discomfort was the other major feature.

**Table 5:** Least acceptable and overall worst part of both investigations according to participant experience questionnaires (numbers represent how many participants provided that particular answer)

	MRI (*n*)	Colonoscopy (*n*)
Least acceptable		
Bowel preparation	4	9
Bowel test	1	0
Other	Back pain Lying on stomach “Nothing”	Pain
No entry	2	0
Overall worst part (free text answer)		
Bowel preparation	1	3
Breath holds	3	N/A
Noise	2	N/A
Discomfort	0	3
Other	Arm uncomfortable Needing to go to the toilet	“Nothing”
No entry	2	3

*MRI* magnetic resonance imaging, *N/A* not applicable

**Fig. 2:**
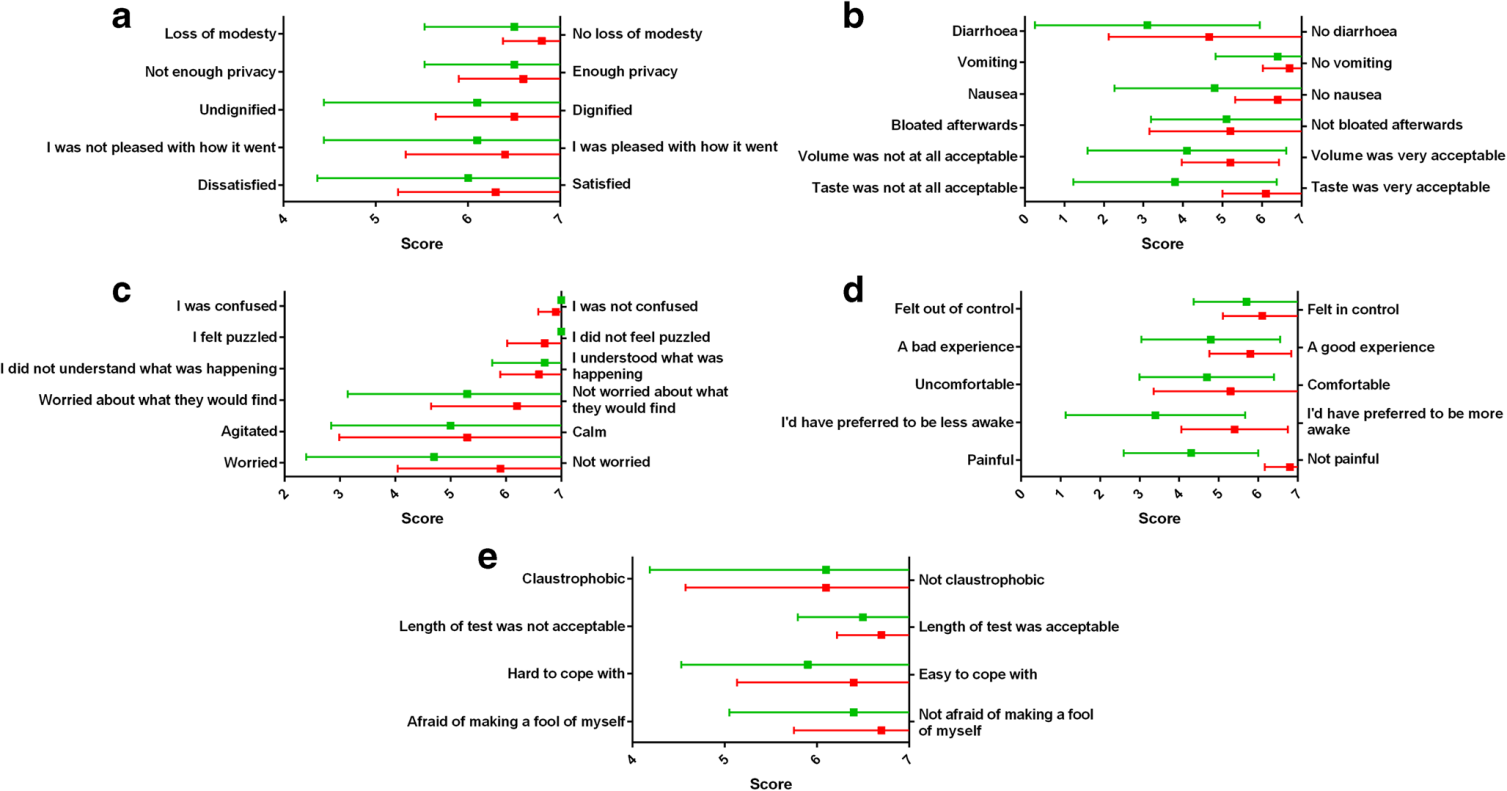
Patient experience questionnaire outcomes. **a**–**e** Results (mean and standard deviation) obtained from patient experience questionnaires for questions common to both investigations. Scores for MRI are represented in red and for colonoscopy in green. The range of scores for each question was from 1 to 7

When asked specific questions relating to the tests (Fig. 2 and Supplementary Fig. 2), there tended to be a preference for MRI over colonoscopy in most areas. The difference in scores was statistically significant for the acceptability of taste of the bowel preparation (score ± SD for MRI 6.1 ± 1.1, for colonoscopy 3.8 ± 2.6, *p* = 0.02), preference for being less awake (score ± SD for MRI 5.4 ± 1.3, for colonoscopy 3.4 ± 2.3, *p* = 0.03), and most notably for pain (score ± SD for MRI 6.8 ± 0.6, for colonoscopy 4.3 ± 1.7, *p* = 0.0004). Colonoscopy only scored (non-significantly) higher than MRI for three questions relating to feeling confused, puzzled, and understanding what was happening (Fig. 2).

**Fig. 3:**
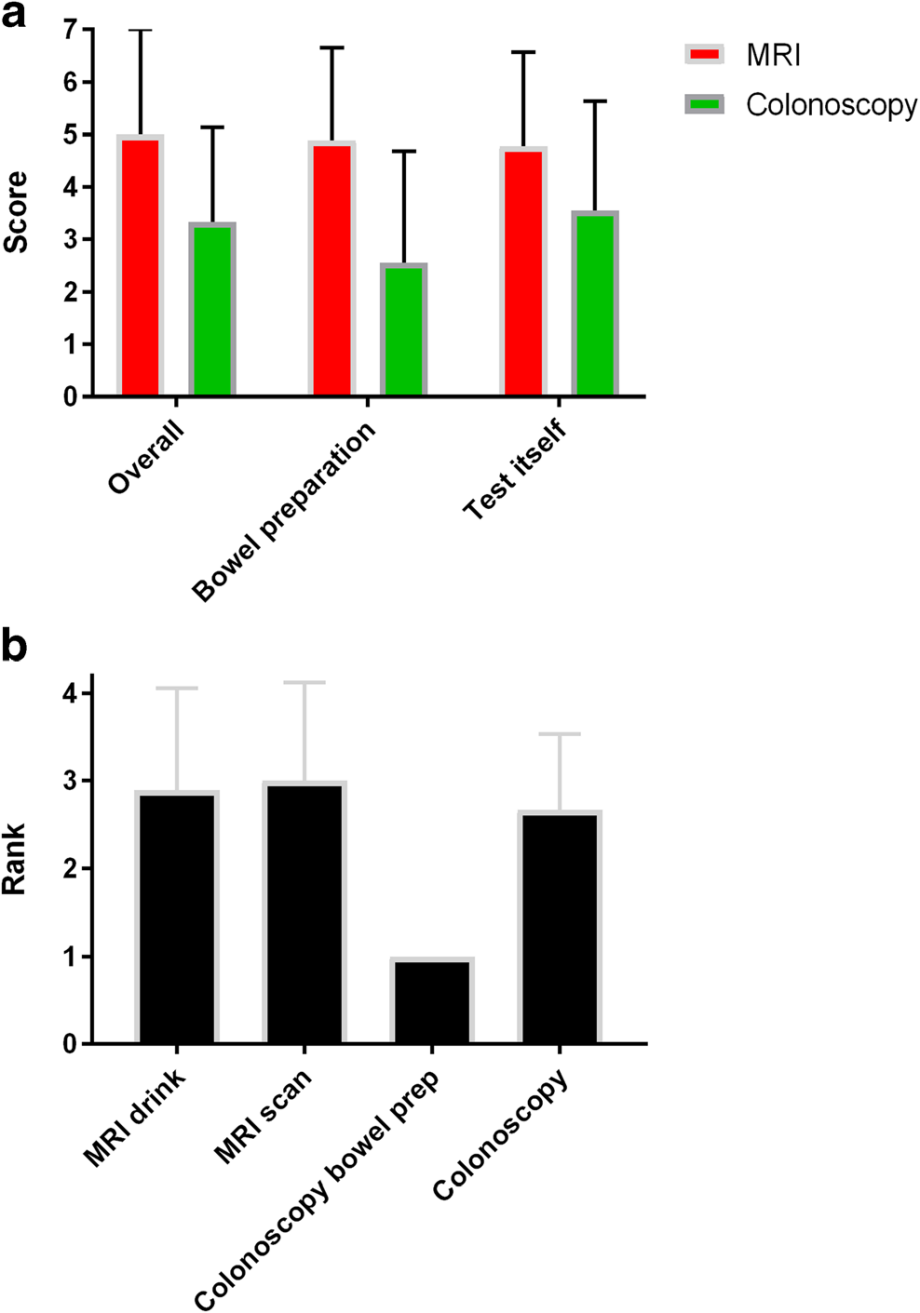
Follow-up questionnaire outcomes. **a** Results (mean and standard deviation) obtained from follow-up questionnaires. Scores for MRI are represented in red and for colonoscopy in green. Participants were asked to evaluate using a 7-point scale (1 = worst, 7 = best) for each investigation overall and for the component parts (bowel preparation and the test itself). **b** Mean and standard deviation of ranks awarded to each of the MRI drink, MRI scan, colonoscopy bowel preparation, and colonoscopy itself on the follow-up questionnaire, starting (i.e., rank = 1) with the worst aspect

We received follow-up questionnaires from nine patients, and results are summarized in Fig. 3. Again, there was an overall preference for MRI over colonoscopy. When asked to rank the various elements of the tests, bowel preparation for the colonoscopy was universally ranked as the worst element. When asked to choose one modality, assuming that a test was essential and that the tests were equivalent in terms of diagnosis and safety, six preferred MRI and three opted for colonoscopy.

### Additional Information Obtained from Each Modality and Correlation with Histological Findings

MRI additionally identified an undescended testis in one individual. Histopathological examination of biopsies obtained during colonoscopy (Supplementary Table S5) from patients with colitis revealed evidence of crypt architectural distortion, cryptitis, and occasionally crypt abscesses or ulcers. Most patients with colitis had evidence of diffuse inflammatory cell infiltrate (acute in patient 1). Pigmented macrophages were common. Where sampled, the terminal ileum was normal except for one patient with pigmented macrophages and one with granulomas. In total, granulomas were seen in four patients, including one without active colitis. Another patient without active colitis had evidence of mild crypt architectural distortion: he was already on mesalazine for a prior history of inflammatory bowel disease.

Overall, there were six bowel segments where both colonoscopy and MRI identified the bowel as normal, but some chronic or quiescent histological changes were noted on biopsy. There were also a further two segments (both in a single patient with definite colitis noted elsewhere in the bowel) where acute histological changes were noted despite normal UCEIS and MRI scores. Conversely, there were four bowel segments where all modalities identified the bowel as colitic, but histology was normal, plus another one segment where only chronic, quiescent histological changes were seen despite abnormal colonoscopy and MRI.

### Fecal Calprotectin Accurately Discriminates Patients with and without Active CGD-Associated Colitis and Demonstrates Better Correlation with Disease Activity than Serum C-Reactive Protein

**Fig. 4:**
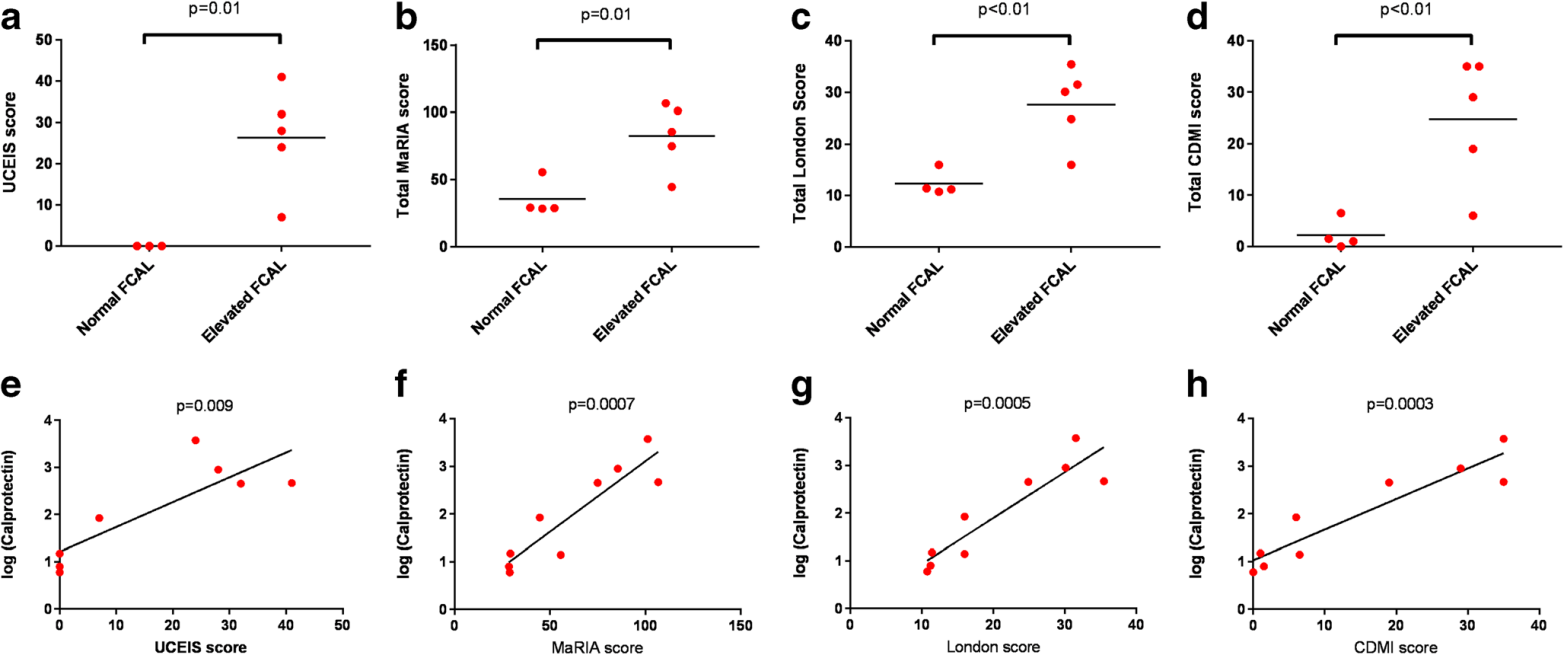
Utility of fecal calprotectin. **a**–**d** Comparison of disease activity scores (endoscopic UCEIS score (**a**), MaRIA score (**b**), London score (**c**), and CDMI score (**d**)) according to whether participants had normal or elevated fecal calprotectin. Analysis is based on the total scores for the entire colon (rectum to caecum) and *p* values are derived from *t* tests. **e**–**h** Correlations between log(fecal calprotectin) and the disease activity scores (endoscopic UCEIS score (**a**), MaRIA score (**b**), London score (**c**), and CDMI score (**d**)). Analysis is based on the total scores for the entire colon (rectum to caecum) and *p* values are derived from Pearson correlation

Fecal calprotectin levels were available from nine participants (in one case, the sample provided was insufficient for analysis). FCP levels elevated above the normal range (> 150 μg/g) were able to accurately differentiate patients with colitis from those without as assessed by total UCEIS (*n* = 8 with both a calprotectin level and a total UCEIS); see Fig. 4a. This equates to 100% sensitivity and 100% specificity in this small cohort. In the patient without a total UCEIS score due to incomplete colonoscopy, there was no colitis seen as far as the splenic flexure (or proximal to this point by MRI) and a normal calprotectin, consistent with other participants’ results. Similarly, the patient with insufficient sample did have endoscopic evidence of colitis (total UCEIS 12), and a subsequent calprotectin level was elevated at 528. There was a significant correlation between UCEIS and log(calprotectin) (*R*^2^ 0.71, *p* = 0.009; Fig. 4e), but not between UCEIS and CRP (*R*^2^ 0.41, *p* = 0.06) or log(CRP) (*R*^2^ 0.42, p = 0.06).

There were also significant differences in MRI scores between patients with elevated or normal FCP (Fig. 4b–d). The discrimination was not perfect due to elevated MRI scores in the patient with abnormal radiological appearances of the rectum not due to active colitis, as described above. However, correlation between MRI scores and log(calprotectin) was strong (for MaRIA *R*^2^ 0.83, *p* = 0.0007; for London score *R*^2^ 0.84, *p* = 0.0005; for CDMI *R*^2^ 0.87, *p* = 0.0003; Fig. 4f–h). The correlations of MRI scores with log(calprotectin) were notably stronger than those with CRP (for MaRIA *R*^2^ 0.50, *p* = 0.02; for CDMI *R*^2^ 0.53, *p* = 0.02; for London score *R*^2^ 0.50, *p* = 0.02) or with log(CRP) (for MaRIA *R*^2^ 0.44, *p* = 0.04; for CDMI *R*^2^ 0.43, *p* = 0.04; for London score *R*^2^ 0.41, *p* = 0.05). The patient with insufficient sample but subsequent elevated calprotectin level had radiological evidence of colitis by all scoring systems.

All participants with elevated FCP had evidence of acute colitis on biopsies, whereas participants with normal FCP had either normal histology or only chronic, quiescent changes.

### The Harvey-Bradshaw Index Does Not Correlate Well with CGD-Associated Colitis Disease Activity

Harvey-Bradshaw index did not differentiate well between patients with and without active colitis as assessed by total UCEIS score or elevated fecal calprotectin (*p* = 0.30), and there was no significant correlation between Harvey-Bradshaw index and total UCEIS score, fecal calprotectin, any of the MRI scores, or CRP.

### Interleukin 12 May Be a Useful Indicator of CGD Colitis Activity

Additional serum samples were available for 9 patients (including all patients with evidence of colitis) in which we measured serum cytokines and markers of immune activation. There were no consistently significant correlations between total UCEIS or MRI scores with IL-1β, TNF, or sCD14. There was a positive correlation between serum IL-6 concentration and the UCEIS score (*R*^2^ 0.67, *p* = 0.01) but not the MRI scores. This discrepancy was attributable to the patient with missing total UCEIS score due to incomplete colonoscopy who had a very elevated level of IL-6 and other innate cytokines despite no evidence of colitis: excluding this patient from correlation analysis with MRI scores resulted in significant (*p* < 0.05) results for all scores.

**Fig. 5:**
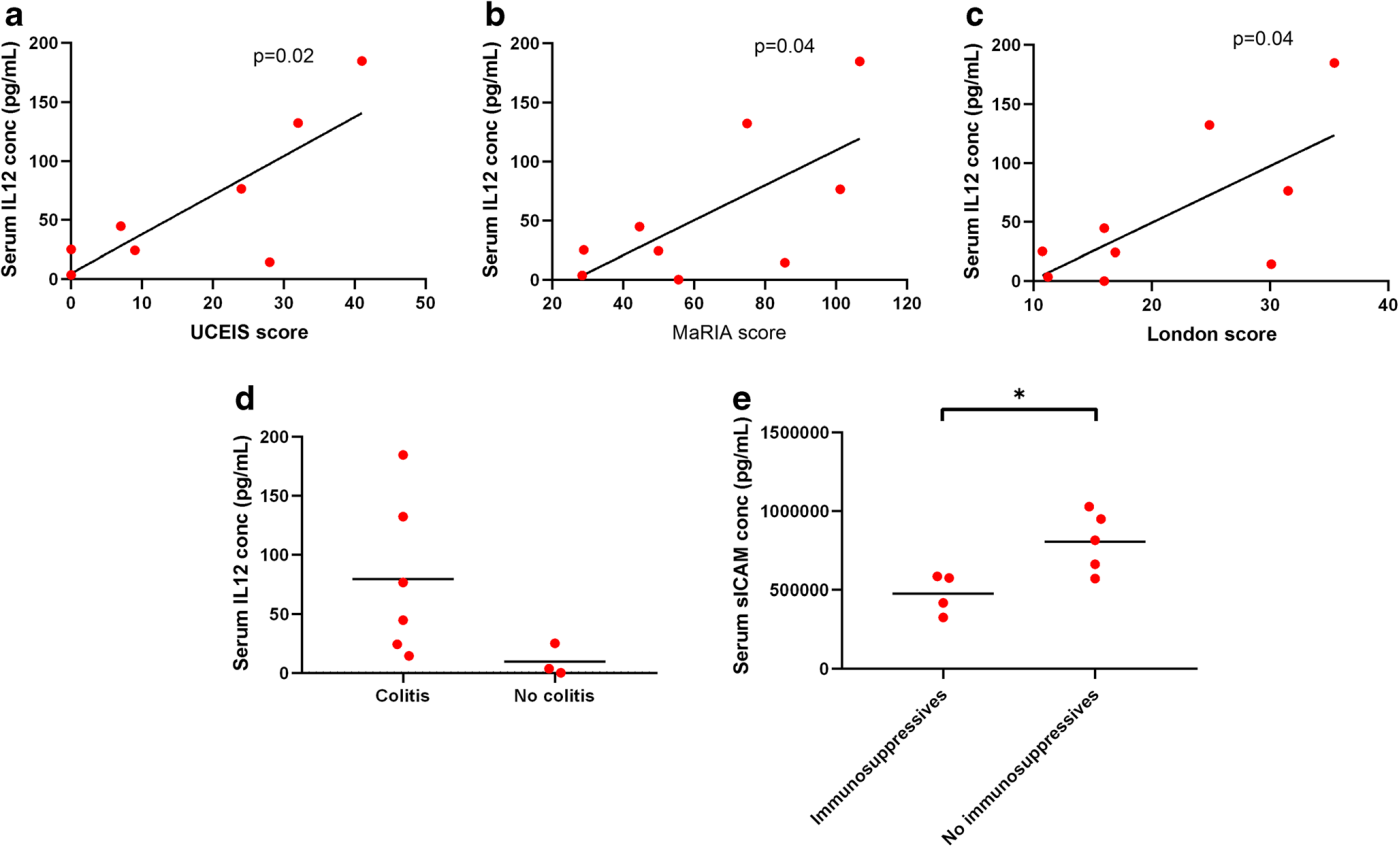
Interleukin 12 level correlates with colitis activity. **a**–**c** Correlations between serum interleukin 12 (IL-12) concentration and the UCEIS score, MaRIA score, and London score. *p* values are derived from Pearson correlation. **d** Serum IL-12 concentration according to presence or absence of colitis. **e** Serum-soluble intercellular adhesion molecule-1 (sICAM) concentration according to presence or absence of immunosuppression (either moderate to high dose corticosteroids and/or azathioprine). *p* value from unpaired *t* test

Serum IL-12 concentration correlated positively with UCEIS score (*R*^2^ 0.65, *p* = 0.02), MaRIA score (*R*^2^ 0.47, *p* = 0.04), and London score (*R*^2^ 0.49, *p* = 0.04), as shown in Fig. 5a–c; concentrations appeared to be consistently low in patients without colitis (Fig. 5d), including in the patient with high innate cytokine levels.

Interestingly, there were significant (*p* < 0.05) negative correlations between serum-soluble ICAM-1 levels with each of the MRI scores. This appears to be explained by the impact of immunosuppression (corticosteroids or azathioprine) which significantly lowered ICAM-1 levels regardless of the presence of severity of colitis (476,865 ± 126,033 pg/ml in patients on immunosuppression versus 805,914 ± 191,230 pg/ml in other patients, *p* = 0.02; Fig. 5e). There were no similar differences in relation to immune suppression for the other serum proteins measured.

## Discussion

We have here demonstrated the importance of non-invasive techniques for diagnosis and monitoring of CGD-associated colitis. MRI assessment of disease activity and log-transformed fecal calprotectin correlated very well with the endoscopic gold standard, while calprotectin could accurately discriminate those with colitis from those without. Serum IL-12 concentration may also be a useful correlate of disease activity and point towards a target for treatment.

Colitis in CGD is important to diagnose and monitor. Although CGD is considered an immune deficiency, severe infectious complications are relatively rare, occurring every few years in patients compliant with prophylactic antimicrobials [15, 16]. In contrast, colitis contributes considerably to a daily symptom burden of living with CGD. Untreated, it can lead to perforation, stricture formation, abscesses, fistulae, and systemic effects including anemia and fatigue [3, 17]. The long-term sequelae, including development of malignancy or systemic complications of chronic inflammation, are not well defined in CGD. However, data from other diseases suggests these are likely to be significant [18]. Colitis activity directs treatment, which is important to titrate so that iatrogenic immunosuppression is kept to a minimum; it is also a major consideration in hematopoietic stem cell transplant decisions and should be well controlled in the peritransplant period.

Colitis has traditionally been assessed via colonoscopy, and this is still considered the gold standard. However, this investigation is invasive and uncomfortable and carries a small risk of serious complications such as perforation [6]. In CGD, information obtained from biopsy is of limited utility because the chronic colitis disease process is similar in all patients and acute infection can usually be diagnosed non-invasively. Indeed, in this study, the pattern of inflammation seen on biopsies (predominantly crypt architectural distortion, cryptitis, and crypt abscess) was consistent between patients. Although one patient on treatment but without evidence of current colitis had evidence of mild crypt architectural distortion and one had a granuloma (which presumably relates to the underlying disease), such findings are unlikely to change management decisions in the absence of visible colitis or elevated fecal calprotectin. It is notable that in two bowel segments (from one patient), acute changes were seen on histological examination despite apparently normal colonoscopy and MRI, but this patient had clear endoscopic and radiological evidence of colitis elsewhere and thus again these biopsies did not alter the diagnosis. There were also four bowel segments where histology was reported as normal despite unanimous macroscopic and radiological evidence of colitis. We suggest that this is likely to represent a sampling error, another potential issue with biopsy as it necessarily assesses only a small portion of the bowel segment. In the absence of transverse colon biopsies, we were unable to undertake a full correlation between histological findings with the results of other investigations.

Imaging techniques, including MRI, have been increasingly demonstrated to be valuable in other inflammatory bowel diseases [7, 8], and scoring systems have been developed [12, 13, 19]. We here demonstrated that MRI-derived scores of colitis activity (“London” score, MaRIA, and CDMI) correlated well with colonoscopy scores (UCEIS). The correlation between radiologists and with colonoscopy scores was best for the London score, although all were highly significant.

Correlation between MRI scores and UCEIS was demonstrated for each bowel segment assessed, albeit there was no unanimous agreement between investigators or modalities. MRI tended to overinterpret findings in the rectum, especially in one patient with a prior history of surgery and radiotherapy, suggesting poorer specificity in this region as previously described [20]. Conversely, MRI appeared somewhat less robust for detecting mild to moderate colitis in the caecum. In all cases where there was discordance, histological findings agreed with colonoscopy supporting this as the gold standard.

Importantly, MRI scored higher than colonoscopy in almost all questions relating to patient preference. Despite a relatively small sample size, the difference in scores was significant for pain, a desire to be less awake, and taste of the bowel preparation drink. Indeed, bowel preparation for colonoscopy scored particularly poorly and was unanimously rated as the worst element in follow-up questionnaires. Of the nine follow-up questionnaires received, six patients preferred MRI over colonoscopy.

MRI was quicker than colonoscopy and was not associated with adverse events (in contrast, one participant suffered presumed bacterial translocation secondary to colonoscopy). Furthermore, imaging was able to assess the entire colon in all patients, while colonoscopy did not reach the terminal ileum in three of ten participants and in one patient only reached the splenic flexure. MRI additionally identified an undescended testis in one participant, while in a subsequent MRI scan assessing CGD colitis (not included in the present study), we also identified pancreatic abnormalities potentially consistent with intraductal papillary mucinous neoplasm (IPMN).

As well as MRI, we also measured fecal calprotectin in participants. Calprotectin, a dimer of the calcium-binding proteins S100A8 and S100A9, is highly represented in neutrophils, and levels in feces are known to reflect disease activity in other inflammatory bowel diseases [21]. We found this to be an excellent discriminator of patients with and without colitis. Although there were only 8 participants with a complete UCEIS score and contemporaneous calprotectin, the remaining 2 patients appeared to maintain the same pattern (i.e., normal FCP in a patient without evidence of colitis as far as the splenic flexure (or more proximally on MRI) and elevated FCP in a subsequent sample from a patient who did have macroscopic colitis). All patients with elevated FCP had histological evidence of colitis, whereas those with normal FCP had either normal histology or chronic quiescent changes only. The FCP result, especially when log-transformed, correlated well with colitis activity. Of note, log(fecal calprotectin) correlated with colitis activity much better than serum C-reactive protein (whether or not log-transformed): this is likely to reflect the fact that patients with CGD can have many other causes of systemic inflammation and concurs with another recent study [10].

Consistent with this conclusion, many serum cytokine levels did not show clear correlations with colitis activity: for example, one participant had very high levels of innate cytokines despite the absence of active colitis. However, IL-12 concentration did correlate with most measures of disease activity. Successful treatment of CGD colitis with the IL-12/23 inhibitor ustekinumab has been described [22], and if our results are replicated in larger cohorts and in studies of gene expression at the disease site, then this treatment target should certainly be further explored. IL-12 may also be useful for disease monitoring, and further prospective studies—perhaps correlating with disease activity as assessed by serial MRI and FCP—would help to clarify this.

The apparent negative correlation between disease activity and serum ICAM-1 levels appeared to be explained by the impact of immunosuppression. This result may nevertheless be important as it suggests that patients with CGD, even in the absence of colitis, may have evidence of endothelial activation with the attendant risk of vascular complications [23]. Regardless of the impact on colitis, broad-spectrum immunosuppression may at least ameliorate this risk. Again, this finding requires further confirmation in a larger group, but a cardiovascular risk phenotype in other chronic inflammatory disorders is well recognized [23] and may become an important source of morbidity in older CGD patients.

Our clinical score based on the Harvey-Bradshaw index did not perform well in predicting colitis activity. There appear to be several explanations for this. “General wellbeing” in CGD may be impacted upon by more than just colitis due to the multisystem nature of the disorder. Interestingly, pain was not a dominant feature, only reported by three patients and never severe. Finally, one participant reported frequent liquid stools despite having normal endoscopic and MRI findings: excluding this participant did result in weak significant correlations (maximal *R*^2^ 0.50) with MRI scores and approaching significant correlation with UCEIS (*R*^2^ 0.50, *p* = 0.06).

Our study had some limitations, most notably the relatively small number of participants. This was of especial importance for cytokine measurements: for example, one patient with significant colitis activity had unusually low results for all measured cytokines which markedly weakened the observed correlations. However, CGD is a rare disorder and, after exclusions, there was a limited potential pool of participants; most patients who declined participation had no symptoms of colitis and would not have had a clinical indication for colonoscopy. Some participants were already on treatment for colitis which would likely have affected endoscopic, MRI, and serum findings, but due to available numbers, it was not possible to restrict the study to patients who were treatment naïve. The study was performed at a tertiary referral center with MRI scans reported by specialist radiologists. This may limit extrapolation to all settings, although CGD patients are usually cared for in specialized services.

In summary, we have demonstrated that CGD-associated colitis can be assessed non-invasively. Fecal calprotectin can be used to confidently exclude active colitis if normal, and the level will give an indication of disease activity. Serum interleukin-12 concentration may also correlate with disease activity and points towards a targeted treatment. Although it may still be important to perform colonoscopy to confirm a diagnosis, and intermittently thereafter, MRI imaging can be used for some monitoring of disease activity. It will be of particular benefit for those unable to tolerate or with contraindications to colonoscopy. While its use in children may be affected by the need for sedation and requirement to ingest a relatively unpalatable drink, these issues equally apply to colonoscopy. In adults, this modality is better tolerated and generally preferred by patients, is quicker, is safer, is more likely to visualize the entire large bowel to terminal ileum, and can identify additional pathologies outside the gastrointestinal tract. We recommend that it is incorporated into routine clinical practice.

## Electronic Supplementary Material

ESM 1 (DOCX 83 kb)
