# Supplementary material for: Chronic Granulomatous Disorder–Associated Colitis Can Be Accurately Evaluated with MRI Scans and Fecal Calprotectin Level
Source: J Clin Immunol. 2019 Jun 6;39(5):494–504. doi: 10.1007/s10875-019-00651-2 (PMC6611758; doi:10.1007/s10875-019-00651-2)
Supplement: Supplementary file 1 — (DOCX 83 kb) [file 10875_2019_651_MOESM1_ESM.docx]

**Supplementary Information**

**Supplementary Appendices**

**Supplementary Appendix 1.** Patient experience questionnaire following MRI scan.

**Patient experience questionnaire for MRI**

**Have you had an MRI before? Yes**

**No**

**What was the least acceptable part of the procedure?**

Bowel preparation

Bowel test

Other

If other, please explain:…………………………………………………………………………

**1. How satisfied were you with the MRI? (On a scale of 1 for the worst to 7 for the best)**

Dissatisfied 1 2 3 4 5 6 7 Satisfied

I was not pleased 1 2 3 4 5 6 7 I was pleased
with how it went with how it went

Undignified 1 2 3 4 5 6 7 Dignified

Not enough 1 2 3 4 5 6 7 Enough privacy

privacy

Loss of 1 2 3 4 5 6 7 No loss of

modesty modesty

**2. How worried were you about the MRI? (On a scale of 1 for the worst to 7 for the best)**

Worried 1 2 3 4 5 6 7 Not worried

Agitated 1 2 3 4 5 6 7 Calm

Worried about what 1 2 3 4 5 6 7 Not worried about

they would find what they would find

I did not understand 1 2 3 4 5 6 7 I understood what what was happening was happening

I felt puzzled 1 2 3 4 5 6 7 I did not feel

puzzled

I was confused 1 2 3 4 5 6 7 I was not confused

**3. How did you find the procedure itself? (On a scale of 1 for the worst to 7 for the best)**

**Bowel preparation**

Taste was not at all 1 2 3 4 5 6 7 Taste was very acceptable acceptable

Volume was 1 2 3 4 5 6 7 Volume was

not at all acceptable very acceptable

Bloated 1 2 3 4 5 6 7 Not bloated afterwards afterwards

Nausea 1 2 3 4 5 6 7 No nausea

Vomiting 1 2 3 4 5 6 7 No vomiting

Diarrhoea 1 2 3 4 5 6 7 No diarrhoea

**MRI**

Painful 1 2 3 4 5 6 7 Not painful

I’d have preferred to 1 2 3 4 5 6 7 I’d have preferred

be less awake to be more awake

Uncomfortable 1 2 3 4 5 6 7 Comfortable

A bad 1 2 3 4 5 6 7 A good experience experience

Felt out 1 2 3 4 5 6 7 Felt in control

of control

Afraid of making 1 2 3 4 5 6 7 Not afraid of making a fool of myself a fool of myself

Uncomfortable lying still 1 2 3 4 5 6 7 Comfortable lying still for for the test the test

Intolerable noise 1 2 3 4 5 6 7 Easy to tolerate

noise

Extremely difficult to 1 2 3 4 5 6 7 Easy to hold

hold breath breath

Hard to cope with 1 2 3 4 5 6 7 Easy to cope with

Length of test was not 1 2 3 4 5 6 7 Length of test was acceptable acceptable

Claustrophobic 1 2 3 4 5 6 7 Not claustrophobic

Overall what was the worst part of the MRI itself (Claustrophobia, breath holds, contrast, anything else?)………………………………………………………………………………………………………….

Comments:…………………………………………………………………...............................................

**For doctor**:

Duration of MRI:…….. minutes

**Supplementary Appendix 2.** Patient experience questionnaire following colonoscopy.

**Patient experience questionnaire for Colonoscopy**

**Have you had a colonoscopy before? Yes**

**No**

**What was the least acceptable part of the procedure?**

Bowel preparation

Bowel test

Other

If other, please explain:…………………………………………………………………………

**1. How satisfied were you with the Colonoscopy? (On a scale of 1 for the worst to 7 for the best)**

Dissatisfied 1 2 3 4 5 6 7 Satisfied

I was not pleased 1 2 3 4 5 6 7 I was pleased with with how it went how it went

Undignified 1 2 3 4 5 6 7 Dignified

Not enough 1 2 3 4 5 6 7 Enough privacy

privacy

Loss of 1 2 3 4 5 6 7 No loss of modesty

modesty

**2. How worried were you about the Colonoscopy? (On a scale of 1 for the worst to 7 for the best)**

Worried 1 2 3 4 5 6 7 Not worried

Agitated 1 2 3 4 5 6 7 Calm

Worried about 1 2 3 4 5 6 7 Not worried about

what they would find what they would find

I did not understood 1 2 3 4 5 6 7 I understood what
what was happening was happening

I felt puzzled 1 2 3 4 5 6 7 I did not feel

puzzled

I was confused 1 2 3 4 5 6 7 I was not confused

**3. How did you find the test itself? (On a scale of 1 for the worst to 7 for the best)**

**Bowel preparation**

Taste was not 1 2 3 4 5 6 7 Taste was very
at all acceptable acceptable

Volume was not 1 2 3 4 5 6 7 Volume was very

at all acceptable acceptable

Bloated 1 2 3 4 5 6 7 Not bloated afterwards afterwards

Nausea 1 2 3 4 5 6 7 No nausea

Vomiting 1 2 3 4 5 6 7 No vomiting

Diarrhoea 1 2 3 4 5 6 7 No diarrhoea

**Colonoscopy**

Painful 1 2 3 4 5 6 7 Not painful

I’d have preferred 1 2 3 4 5 6 7 I’d have preferred to to be less awake be more awake

Uncomfortable 1 2 3 4 5 6 7 Comfortable

A bad 1 2 3 4 5 6 7 A good experience

experience

Felt out 1 2 3 4 5 6 7 Felt in control

of control

Afraid of 1 2 3 4 5 6 7 Not afraid of making making a fool of myself a fool of myself

Uncomfortable 1 2 3 4 5 6 7 Comfortable moving moving for the test for the test

Hard to cope 1 2 3 4 5 6 7 Easy to cope with

with

Length of test 1 2 3 4 5 6 7 Length of test was was not acceptable acceptable

Claustrophobic 1 2 3 4 5 6 7 Not claustrophobic

Overall what was the worst part of the colonoscopy itself (discomfort, injections, moving, anything else?)………………………………………………………………………………………………………………

Comments:………………………………………………………………….....................................................

**For doctor**:

Duration of colonoscopy:…….. minutes

Colonoscopy was done using sedation/analgesics: Yes No

**Supplementary Appendix 3.** Follow-up questionnaire one week after investigations.

**After both investigations**

**How unpleasant or pleasant was the MRI? (On a scale of 1 for the worst to 7 for the best) i.e. if it was awful 1, if it was very pleasant 7**

Overall
1 2 3 4 5 6 7

The oral drink (and its effects)
1 2 3 4 5 6 7

The scan itself
1 2 3 4 5 6 7

**How unpleasant or pleasant was the colonoscopy? (On a scale of 1 for the worst to 7 for the best) i.e. if it was awful 1, if it was very pleasant 7**

Overall
1 2 3 4 5 6 7

The bowel preparation (and its effects)
1 2 3 4 5 6 7

The colonoscopy itself
1 2 3 4 5 6 7

**Please rank these from 1 to 4, listing the MOST unpleasant first**

MRI drink (and its effects)

MRI scan

Bowel preparation for colonoscopy (and its effects)

Colonoscopy

**If we suppose that for medical reasons a diagnostic examination of your bowel is essential but you can choose between MRI and colonoscopy. Assuming that both studies are equivalent in terms of diagnosis and safety, which of these examinations would you choose?**

MRI

Colonoscopy

No preference

**Supplementary Appendix 4**

**Additional definitions for MRI scoring systems**

| **Definitions for the qualitative CDMI score** | | | | |
| --- | --- | --- | --- | --- |
| **Score** | **0** | **1** | **2** | **3** |
| Mural thickness | 1-3 mm | >3-5 mm | >5-7 mm | >7 mm |
| Mural T2 signal | Equivalent to normal bowel wall | Minor increase in signal - bowel wall appears dark grey on fat saturated images | Moderate increase in signal - bowel wall appears light grey on fat saturated images | Marked incease in signal - bowel wall contains areas of white high signal approaching that of luminal content |
| Perimural T2 signal | Equivalent to normal mesentery | Increase in mesenteric signal but no fluid | Small fluid rim (≤2 mm) | Larger fluid rim (>2 mm) |
| Enhancement | Equivalent to normal bowel wall | Minor enhancement - bowel wall signal greater than normal small bowel but significantly less than nearby vasular structures | Moderate enhancement - bowel wall signal increased but somewhat less than nearby vascular structures | Marked enhancement - bowel wall signal approaches that of nearby vascular structures |
|  | | | | |

**Definitions for the MaRIA score**

Relative contrast enhancement (RCE) = [(WSI postgadolinium – WSI pregadolinium)/(WSI pregadolinium)] × 100 × (SD noise pregadolinium / SD noise postgadolinium)

Where:

WSI = wall signal intensity at 70 seconds measured in VIBE T1‐weighted images, performed in a region of interest from the areas with most thickening (score is average of three WSI measurements)

SD noise pregadolinium = average of three SD of the signal intensity measured outside of the body before gadolinium injection

SD noise postgadolinium = SD of the same noise after gadolinium administration.

**Supplementary Tables**

**Supplementary Table S1.** Inclusion and exclusion criteria for study.

| Eligibility criteria | - Patients with a known diagnosis of CGD - Able to provide informed consent - Aged 18 to 70 years |
| --- | --- |
| Exclusion criteria | - Pregnancy - Contraindication to MRI (eg pacemaker, metallic implant, severe claustrophobia) - History of adverse reaction to any of the medications used as part of the procedures, or previous adverse event following colonoscopy - Active infection of gastrointestinal tract or significant active infection elsewhere - On anticoagulant medication or any known bleeding disorder - Previous colectomy or hemi-colectomy or presence of colostomy or defunctioning ileostomy |

**Supplementary Table S2.** Summary of scores and results from study.

| **ID** | **Harvey Bradshaw Index (total)** | **UCEIS score** | **MaRIA score** | **CDMI score** | **London score** | **CRP (mg/L)** | **Faecal calprotectin (mcg/g)** | **IL1b (pg/mL)** | **IL6 (pg/mL)** | **IL12 (pg/mL)** | **TNF (pg/mL)** | **sICAM (pg/mL)** | **sCD14 (pg/mL)** |
| --- | --- | --- | --- | --- | --- | --- | --- | --- | --- | --- | --- | --- | --- |
| **1** | 9 | 24 | 101.222 | 35 | 31.53 | 42 | 3760 | 24.88 | 350.16 | 76.48 | 6.21 | 326355.6 | 2944500 |
| **2** | 7 | 32 | 74.91942 | 19 | 24.87 | 28 | 451 | 60.29 | 322.76 | 132.26 | 3.03 | 571253.1 | 6512400 |
| **3** | 7 | 0 | 29.2429 | 1 | 11.41 | 2 | 15 | - | - | - | - | - | - |
| **4** | 1 | 28 | 85.578 | 29 | 30.1 | 64 | 896 | 3.81 | 54.56 | 14.43 | 0.4 | 419304.1 | 5376100 |
| **5** | 9 | 41 | 106.6757 | 35 | 35.445 | 27 | 466 | 39.88 | 528.48 | 184.6 | 6.19 | 575927.8 | 5569900 |
| **6** | 2 | 9 | 49.90731 | 9.5 | 16.91 | 32 | Insufficient | 5.3 | 50.36 | 24.21 | 1.33 | 816196.7 | 6166700 |
| **7** | 2 | 7 | 44.59736 | 6 | 15.97 | 3 | 84 | 12.96 | 73.71 | 44.83 | 1.99 | 662852.2 | 6693300 |
| **8** | 2 | 0 | 28.859 | 0 | 10.74 | 13 | 6 | 8.25 | 60.65 | 25.01 | 0 | 585829.9 | 6463300 |
| **9** | 3 | 0 | 28.4242 | 1.5 | 11.21 | 13 | 8 | 4.77 | 22.11 | 3.53 | 2.15 | 1029200 | 4218400 |
| **10** | 0 | 0 | 55.59159 | 6.5 | 15.97 | 27 | 14 | 40.05 | 766.93 | 0 | 13 | 950067.3 | 5423500 |

UCDEIS, Ulcerative Colitis Endoscopic Index of Severity; MaRIA, Magnetic Resonance Index of Activity; CDMI, Crohn’s Disease MRI Index; CRP, C-Reactive protein; mg, milligramme; L, litre; mcg, microgramme; g, gramme; IL, interleukin; pg, picogramme; mL, millilitre; TNF, tumour necrosis factor; sICAM, soluble intercellular adhesion molecule; CD, cluster of differentiation.

Note that scores from UCEIS, MaRIA, CDMI and London relate to colon only (rectum to caecum), excluding terminal ileum.

**Supplementary Table S3**. Clinical features of inflammatory bowel disease.

| **ID** | **General well-being**  **[0 = very well**  **1 = slightly below average**  **2 = poor**  **3 = very poor**  **4 = terrible]** | **Abdominal pain**  **[0 = none**  **1 = mild**  **2 = moderate**  **3 = severe]** | **Number of liquid stools per day** | **Abdominal mass**  **[0 = none**  **1 = dubious**  **2 = definite**  **3 = tender]** | **Perianal disease**  **[1 point for each fistula, fissure or abscess]** |
| --- | --- | --- | --- | --- | --- |
| **1** | 1 | 2 | 6 | 0 | 0 |
| **2** | 1 | 0 | 5 | 0 | 1 |
| **3** | 0 | 2 | 5 | 0 | 0 |
| **4** | 0 | 0 | 0 | 0 | 1 |
| **5** | 4 | 2 | 3 | 0 | 0 |
| **6** | 1 | 0 | 0 | 0 | 1 |
| **7** | 0 | 0 | 0 | 0 | 2 |
| **8** | 2 | 0 | 0 | 0 | 0 |
| **9** | 2 | 0 | 0 | 0 | 1 |
| **10** | 0 | 0 | 0 | 0 | 0 |

**Supplementary Table S4.** Detail of endoscopic findings with reference to the Ulcerative Colitis Endoscopic Index of Severity (UCEIS) score.

| ID | UCEIS Vascular pattern  [0=normal, 1=patchy loss, 2=obliterated] | | | | | | | UCEIS Bleeding  [0=none, 1=mucosal, 2=luminal mild, 3=luminal severe] | | | | | | | UCEIS erosions and ulcers  [0=normal, 1=erosions, 2=superficial ulcer, 3=deep ulcer] | | | | | | |
| --- | --- | --- | --- | --- | --- | --- | --- | --- | --- | --- | --- | --- | --- | --- | --- | --- | --- | --- | --- | --- | --- |
|  | Rectum | Sigmoid | Splenic flexure | Trans-verse | Hepatic flexure | Caecum | Terminal ileum | Rectum | Sigmoid | Splenic flexure | Trans-  verse | Hepatic flexure | Caecum | Terminal ileum | Rectum | Sigmoid | Splenic flexure | Trans-  verse | Hepatic flexure | Caecum | Terminal ileum |
| 1 | 2 | 2 | 2 | 0 | 0 | 0 | 0 | 3 | 3 | 3 | 0 | 0 | 0 | 0 | 3 | 3 | 3 | 0 | 0 | 0 | 0 |
| 2 | 2 | 2 | 2 | 0 | 1 | 1 | 0 | 3 | 3 | 3 | 0 | 1 | 1 | 0 | 3 | 3 | 3 | 0 | 2 | 2 | 0 |
| 3 | 0 | 0 | 0 | 0 | 0 | 0 | 0 | 0 | 0 | 0 | 0 | 0 | 0 | 0 | 0 | 0 | 0 | 0 | 0 | 0 | 0 |
| 4 | 1 | 1 | 2 | 2 | 1 | 1 | 0 | 1 | 1 | 2 | 2 | 1 | 2 | 0 | 1 | 2 | 3 | 3 | 1 | 1 | 0 |
| 5 | 2 | 2 | 2 | 2 | 2 | 2 | N/A | 2 | 1 | 2 | 2 | 2 | 2 | N/A | 3 | 3 | 3 | 3 | 3 | 3 | N/A |
| 6 | 2 | 1 | 0 | 0 | 0 | 1 | 1 | 1 | 0 | 0 | 0 | 0 | 1 | 1 | 2 | 1 | 0 | 0 | 0 | 0 | 1 |
| 7 | 1 | 1 | 1 | 0 | 0 | 0 | N/A | 0 | 1 | 1 | 1 | 0 | 0 | N/A | 0 | 1 | 0 | 0 | 0 | 0 | N/A |
| 8 | 0 | 0 | 0 | 0 | 0 | 0 | 0 | 0 | 0 | 0 | 0 | 0 | 0 | 0 | 0 | 0 | 0 | 0 | 0 | 0 | 0 |
| 9 | 0 | 0 | 0 | 0 | 0 | 0 | 0 | 0 | 0 | 0 | 0 | 0 | 0 | 0 | 0 | 0 | 0 | 0 | 0 | 0 | 0 |
| 10 | 0 | 0 | 0 | N/A | N/A | N/A | N/A | 0 | 0 | 0 | N/A | N/A | N/A | N/A | 0 | 0 | 0 | N/A | N/A | N/A | N/A |

N/A, not applicable.

**Supplementary Table S5.** Summary of histological findings (biopsies taken from rectum, sigmoid, splenic flexure, hepatic flexure and caecum).

| **ID** | **Crypt architectural distortion** | **Cryptitis** | **Crypt abscesses** | **Inflammatory cell infiltrate** | **Granulomas** | **Pigmented macrophages** | **Ulceration** |
| --- | --- | --- | --- | --- | --- | --- | --- |
| 1 | R, Si, Sp | R, Si, Sp | - | R, Si, Sp | - | H, C | R, Si |
| 2 | R, Si, C | R, Si, H | C | R, Si, H, C | - | - | - |
| 3 | Si, Sp, H, C | - | - | - | - | Throughout | - |
| 4 | Si, Sp, H, C | H | - | Sp, C | Si, H | Throughout | Sp |
| 5 | R, Sp, H, C | - | R | Throughout | - | - | - |
| 6 | R, Si, Sp, C | H, C | R, Si, C | R, Si, Sp, C | R, Si, Sp | - | R |
| 7 | - | Sp | - | - | Sp, H | Throughout | - |
| 8 | - | - | - | - | Sp | Throughout | - |
| 9 | - | - | - | - | - | Throughout | - |
| 10 | - | - | - | - | - | Throughout | - |

R, rectum; Si, sigmoid; Sp, splenic flexure; H, hepatic flexure; C, caecum.

**Supplementary Figures**

**Supplementary Figure S1.** Mean and standard deviation of results from all participants for UCEIS (A), MaRIA score (B), London score (C) and CDMI score (D) according to bowel segment.

D

C

B

A

**Supplementary Figure S2.** Results (mean and standard deviation) obtained from patient experience questionnaires on questions relating solely to MRI (A) or colonoscopy (B).

A

B

B
